# Prevalence and determinants of depressive symptoms among postpartum mothers visiting rural health centre in Ethiopia: A cross-sectional study

**DOI:** 10.4102/jphia.v16i4.1576

**Published:** 2025-12-18

**Authors:** Abigael Abiy Mesfin, Abdurahman Yimer, Abraham Begashaw, Amir Nigusu, Amesiyas Zewude, Amanuel Assefa, Abraham Genetu Tiruneh, Samuel Mesfin Girma

**Affiliations:** 1 Science and Innovation Directorate, Africa Centres of Disease Control and Prevention, Addis Ababa, Ethiopia; 2 Department of Surgery, Addis Ababa University, Addis Ababa, Ethiopia

**Keywords:** postpartum, depression, associated factors, rural, Ethiopia, prevalence, magnitude, health centre

## Abstract

**Background:**

Postpartum depression (PPD) is a form of major depression that occurs after childbirth and may begin during pregnancy. It affects 10% – 20% of new mothers globally and has a relatively high prevalence in developing countries. Postpartum depression can negatively impact maternal health, child development and family well-being. In Ethiopia, there is limited evidence from the Oromia Region despite its large population.

**Aim:**

This study aimed to assess the magnitude and associated factors of PPD among mothers attending the Batu Health Center.

**Setting:**

The study was conducted from 11 July 2022 until 11 August 2022 in Batu, East Shewa Zone, Oromia, a regional state of Ethiopia.

**Methods:**

A facility-based cross-sectional study was conducted among postnatal mothers who gave birth within the past 12 months. A total of 195 mothers were selected via simple random sampling. The data were analysed via Statistical Package for Social Sciences (SPSS) version 20. Bivariable and multivariable logistic regressions were conducted to identify factors associated with PPD at a significance level of *p* < 0.05.

**Results:**

The prevalence of postpartum depression among the participants was 24.6%. Among those with PPD, 18.75% had thoughts of self-harm. The factors significantly associated with PPD included a history of abortion (adjusted odds ratio [AOR] = 2.574; 95% confidence interval [CI]: 1.320–5.022), a history of mental illness of the mother (AOR = 7.836; 95% CI: 3.077–12.648) and low social support (AOR = 9.325; 95% CI: 5.849–12.801).

**Conclusion:**

This study revealed a high prevalence of postpartum depression among mothers in Batu.

**Contribution:**

Routine screening, health professional training and public awareness efforts are essential to improve early detection and support for affected mothers in Ethiopia.

## Background

Postpartum depression is a severe type of depression that some new mothers endure. Because it can begin during pregnancy and persist after childbirth, it is sometimes referred to as ‘peripartum depression’. Worldwide, an average of 10% to 20% of mothers experience symptoms of depression after giving birth, and multiple factors contribute to these symptoms.^1^ In developed countries, the prevalence of postpartum depression (PPD) is thought to be approximately 10%,^2^ whereas it is estimated to be approximately 20% in developing nations.^3^ A systematic review that included 19 studies from all over Africa revealed that the prevalence of postpartum depression was 16.84%.^4^ Even though it is unlikely to be the sole contributor, the change in progesterone levels is mostly responsible for these emotions.^5^ Symptoms of postpartum depression can include strong mood swings, depressed mood, crying excessively, having a hard time bonding with one’s child, withdrawing from relatives and friends, sleep disturbances, excessive fatigue or lack of energy, loss of enjoyment from previously enjoyed activities, severe irritation and considering oneself a bad mother.^6^

Social stresses such as poverty, intimate partner abuse, a history of miscarriage and unwanted pregnancy are risk factors for postpartum depression, and these factors inevitably have a negative impact on maternal health.^7^ The mother and her children may suffer from long-term negative effects of postpartum depression if left untreated. Chronic depression in postnatal women may be a factor contributing to subsequent interpersonal, behavioural, cognitive and emotional challenges.^8^ Postnatal depression places a heavy strain on spouses and members of the immediate family, limiting social and leisure activities and leading to financial issues for the family.^9,10,11^ Compared with women without postnatal depression, women with postnatal depression are more likely to have unhealthy lifestyles, which include poor nutrition and sleep habits.^11,12,13^ Mothers suffering from postpartum depression may have a harder time interpreting and reacting to the cues sent by the baby.^14^ The effect of postpartum depression is not limited to mothers. Children who are taken care of by mothers who suffer from postpartum depression are also victims. Postnatal depression has been found to be associated with underweight status, stunting and decreased mental development.^15^ Breastfeeding cessation occurs earlier in depressed mothers than in mothers who are not depressed.^16,17,18^ Despite the burden, Ethiopia and other developing nations lack access to necessary medical care.^19^

Although studies have been conducted in different parts of Ethiopia, very few have been conducted in the Oromia Region. Oromia occupies Ethiopia’s largest land area and has the highest number of inhabitants among all the regional states of Ethiopia.^20^ Nonetheless, only one study was performed in the region on the topic of postpartum depression. This approach has the potential to under-represent the region on national and international datasets. Hence, this research aims to address this gap and contributes to building strong and more representative evidence for postpartum depression in Ethiopia. This study aimed to assess the magnitude and factors associated with postpartum depression among mothers visiting the Batu Health Center.

## Research methods and design

### Study area and period

Batu is located in the centre of the Ethiopian Rift Valley and is one of the 46 towns of the Oromia Region in Ethiopia, with an average elevation of 1657 m above sea level. It is located 165 km south of Addis Ababa. The town’s population increased from 49 416 in 2000 to 78 784 (40 180 men and 38 604 women) in 2018.^21^

The government-owned health facilities in Batu include one hospital and two health centres. This study is a health facility-based cross-sectional study of postnatal mothers visiting the Batu Health Center. Batu health centres are expected to serve 41 381 people in Batu. The study was conducted from 11 July 2022 until 11 August 2022 in Batu, East Shewa Zone, Oromia, a regional state of Ethiopia.

### Study design

A cross-sectional study design was used among postnatal women who gave birth within 12 months prior to the date of data collection.

### Inclusion criteria

All mothers who gave birth at least 2 weeks before the interview and who were residents of the study area for at least 6 months.

### Exclusion criteria

- Mothers who gave birth 12 months or more before the data collection period.
- Mothers who were sick and were not able to respond to the questions asked.

### Sample size and sampling technique

Considering the average prevalence rate to be 22% by Duko et al.^22^ and a margin of error of 5% and 95% confidence interval (CI), the sample size was initially calculated to be 264:


$$\begin{array}{l} n=(1.96{)}^{2}\times 0.22\times 0.78\;(0.05{)}^{2}\\ n=263.68(264) \end{array}$$ [Eqn 1]

On average, 750 mothers visit the health centre each month. Based on an initial calculated sample size of 264 and a total population of 750 for the 1-month study period, application of the finite population correction (FPC) factor yielded an adjusted sample size of 195:


$$\frac{nc=nxN.}{\left(N+(n-1)\right)}$$ [Eqn 2]

*n* = Sample Size

*N* = Population size

*n*_c_ = Corrected sample size

*n*_c_ = 195

Adding a 10% non-response rate, the final sample size was 215. Simple random sampling was used as the sampling procedure, with a random number generator used to select mothers to be interviewed from vaccination attendance lists.

### Data collection tool and procedures

Data were collected via a structured questionnaire that was administered by interviewers. Six 5th-year medical students at Tikur Anbessa Specialized Hospital collected data through face-to-face individual interviews with mothers, and the responses were entered into Google Forms. The Edinburgh Postpartum Depression Scale (EPDS) was used to assess PPD. The EPDS is a 10-item self-report measure that is intended to screen for PPD and is based on a 1-week recall. The EPDS includes a question on whether the mothers had the intention of self-harm. The EPDS was validated as a screening tool to identify postnatal depression symptoms in Ethiopia, as its sensitivity was 78.9% and its specificity was 75.3%.^20,21^ Social support was assessed via the Maternal Social Support Scale (MSSS). The MSSS was developed by Webster and colleagues,^23^ and a maximum score of 30 was obtainable, with each item measured out of five on a Likert scale.

### Data processing and analysis

The data were imported from Google Forms, cleaned and checked for completeness, accuracy and missing data. The data were then exported to Statistical Package for Social Sciences (SPSS) version 20 for analysis. Bivariable and multivariable logistic regression analyses were used to determine the associations of postpartum depressive symptoms with socio-demographic, obstetric and psychosocial factors. The variables studied were initially entered into bivariable logistic regression. A cutoff of *p*-value = 0.2 was used to screen variables with potential association. Variables that qualified in bivariable logistic regression were further entered into multivariable logistic regression to control for confounders. Significance was determined at a *p*-value < 0.05.

### Operational definition

- Postpartum depression: a form of major depression that begins after 2 weeks of delivery^5^ and within 12 months of childbirth^24^ diagnosed with a score of ≥ 11 on the EPDS.
- Low social support: score of < 18 on the MSSS.
- Medium social support: score of 18–23 on the MSSS.
- High social support: score of 24–30 on the MSSS.

### Variables

#### Dependent variable: Postpartum depression

Independent variables: age, occupation, marital status, number of pregnancies, abortion history, pregnancy desirability, mode of delivery, complication during delivery, postnatal care visit, history of mental illness of the mother and availability of social support.

### Ethical considerations

Ethical clearance to conduct this study was obtained from the Addis Ababa University College of Health Sciences School of Medicine Research Ethics Committee (No. 02/22/15). The study was conducted in accordance with the ethical principles outlined in the Declaration of Helsinki. Informed written consent was obtained from all the respondents prior to their participation in the study. Confidentiality was maintained, and the data were collected anonymously. All data collected during the study were stored securely to ensure confidentiality and data integrity.

## Results

### Socio-demographic characteristics of the study participants

With a 90.7% response rate, a total of 195 postpartum mothers were included in our study, and the majority (73.3%) were between the ages of 21 years and 30 years. A total of 68.7% of the participants were housewives, whereas the others were merchants, farmers, daily labourers, government employees and others. Approximately 98.5% were married, as described in Table 1.

**TABLE 1:** Socio-demographic characteristics of the participants.

Characteristic	Category	*n*	%
Occupation	Housewife	134	68.7
	Government employee	23	11.8
	Merchant	17	8.7
	Student	4	2.1
	Farmer	7	3.6
	Others	10	5.1
Marital status	Married	192	97.9
	Divorced	2	1.0
	Single	1	0.5
Age group (years)	16–20	30	15.4
	21–25	72	36.9
	26–30	71	36.4
	≥ 31	22	11.3

### Obstetric history of the study participants

Half (51.3%) of the participants had been pregnant once or twice. The majority (82.6%) of the respondents had no abortion history, and 16.4% had a history of at least one abortion. A total of 87.7% of the respondents said that their recent pregnancy was planned. A total of 90.8% of the participants had antenatal care (ANC) visits during their pregnancy. Among the mothers who had ANC visits, 88.2% were not advised about PPD, as shown in Table 2.

**TABLE 2:** Obstetric characteristics of the study participants.

Variable	Category	*n*	%
Number of pregnancies	1–2 pregnancies	100	51.3
	> 2 pregnancies	95	48.7
History of abortion	No	161	82.6
	Yes (≥ 1 abortion)	34	17.4
Pregnancy desirability	Yes	171	87.7
	No	24	12.3
Had antenatal care (ANC) visits	Yes	177	90.8
	No	18	9.2
Among ANC attendees (*n* = 177), advised about PPD	No	156	88.2
	Yes	21	11.8
Mode of delivery	Vaginal (spontaneous/assisted)	179	91.8
	Caesarean section	16	8.2
Complications during delivery	No	179	91.8
	Yes	16	8.2
Scheduled or attending PNC visit	No	182	93.3
	Yes	13	6.7

ANC, antenatal care; PNC, postnatal care; PPD, postpartum depression.

A total of 91.8% of the respondents had a vaginal delivery (both spontaneous and assisted vaginal deliveries), whereas 8.2% had a caesarean section. The majority (91.8%) of the patients delivered without complications. Approximately 93.3% of the study participants had no impending PNC visit.

### Psychosocial history of the postpartum mothers in our study

Among the respondents, 73.8% had no infant health problems. Seventy-nine per cent of the mothers had children of the desired sex, as shown in Table 3. A total of 97.9% of the participants did not have a history of mental illness.

**TABLE 3:** Psychosocial characteristics of the study participants.

Characteristic	Category	*n*	%
Infant health problems	No	144	73.8
	Yes	51	26.2
Child of desired gender	Yes	154	79.0
	No	41	21.0
History of mental illness of the mother	No	191	97.9
	Yes	4	2.1

### Prevalence of postpartum depression

**FIGURE 1:**
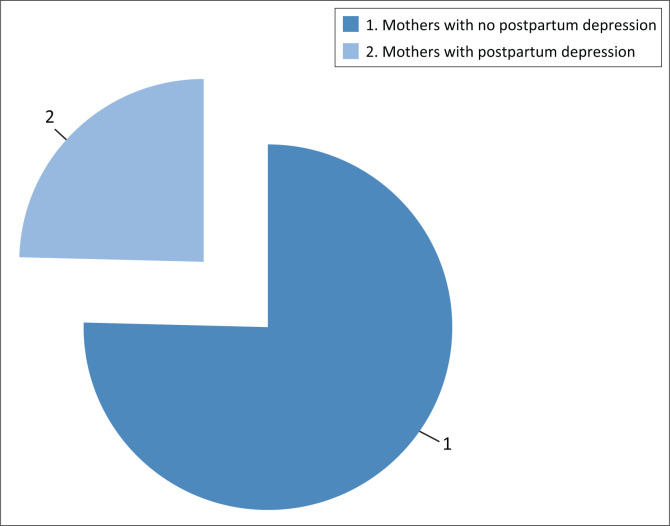
Prevalence of postpartum depression among mothers attending Batu Health Center, Oromia, Ethiopia (*N* = 195).

Currently, a cutoff score of 11 is recommended, which seems to maximise test performance in terms of both sensitivity and specificity.^24^ According to the cutoff points of the EPDS (≥ 11), 24.6% of the total respondents had postpartum depression, whereas 75.4% did not have depressive symptoms, as shown in Figure 1. In the assessment with the EPDS, among the mothers who had postpartum depression, 18 (24.62%) had thoughts of self-harm.

Among all the mothers who had participated in our study, 77.4% had never heard of PPD before. Among the mothers who had postpartum depression, the majority (85.4%) had not even heard about the disease before.

### Social support

An MSSS was used to determine the availability of social support. Among the participants in the study, 9.2% had low social support. Among those mothers who had postpartum depression, 25% had low social support.

Among the study participants who had postpartum depression, 18.75% reported that they did not ask for help from their family in times of need, 8.3% did not agree that their family friend network was good and 8.3% were not completely assured that they could completely rely on their spouse. A total of 54.34% had conflicts with their spouse, 14.9% felt controlled by their family and 18.75% did not feel loved and accepted by their family.

### Factors associated with postpartum depression

Bivariable logistic regression was initially used to assess the associations between postpartum depression and other factors. A cutoff of 0.2 was used to screen for variables with potential associations. Variables that qualified for the bivariable logistic regression were entered into the multivariable regression. A *p*-value of 0.05 or less was used to determine significant associations.

There was no association between age, occupation or marital status and postpartum depression. In the bivariable analysis, a significant association was found between abortion history, parity, the presence of complications during delivery, pregnancy desirability, a history of mental illness of the mother and a lack of social support with postpartum depression, as shown in Table 4.

**TABLE 4:** Bivariable logistic regression analysis of variables.

Variables	*p*	COR	95% CI
Age	0.875	0.99	0.92–1.07
**Occupation**	-	-	-
Daily labourer	0.661	2.00	0.09–44.35
Farmer	0.819	1.33	0.11–15.70
Government	0.253	0.28	0.03–2.50
Housewife	0.269	0.32	0.04–2.39
Merchant	0.194	0.21	0.02–2.19
Marital status	0.745	-	-
Parity (no. of pregnancy)	**0.089**	**1.18**	**0.98–1.43**
Abortion	**0.006**	**2.57**	**1.32–5.02**
Pregnancy desirability	**0.020**	**0.35**	**0.15–0.85**
**Mode of delivery**	-	-	-
Caesarean section	0.257	1.86	0.64–5.43
Vaginal delivery	0.691	1.33	0.33–5.37
Complications during delivery	**0.197**	**0.49**	**0.16–1.45**
History of mental illness of the mother	**0.001**	**9.21**	**4.06–14.33**
Lack of social support	**0.001**	**10.12**	**6.62–13.63**

Note: Data in bold indicate significant association.

COR, crude odds ratio; CI, confidence interval.

The odds of developing postpartum depression among mothers with a history of abortion were 2.5 times higher (adjusted odds ratio [AOR] = 2.57; 95% CI: 1.32–5.02) as compared with those mothers without a history of abortion. The odds of developing postpartum depression among mothers with a history of mental illness were nearly eight times higher as compared with mothers without such a history (AOR = 7.863; 95% CI: 3.077–12.648). A lack of social support increased the likelihood of developing postpartum depression by almost nine times, as shown in Table 5.

**TABLE 5:** Multivariate logistic regression of associated factors.

Variables	*p*	AOR	95% CI
Number of pregnancy	0.638	1.05	0.85, 1.31
Abortion history	**0.006**	**2.57**	**1.32, 5.02**
Pregnancy desirability	0.076	0.43	0.17, 1.09
Complications during delivery	0.213	2.00	0.67, 5.95
History of mental illness of the mother	**0.001**	**7.86**	**3.08, 12.65**
Lack of social support	**0.001**	**9.32**	**5.85, 12.80**

Note: Data in bold indicate significant association.

CI, confidence interval.

## Discussion

This study revealed that the magnitude of 24.6% was high. This result aligns with Ethiopia’s pooled postpartum depression prevalence of 22.89%.^25^ A similar health facility-based study in Debre-Berhan, Ethiopia, reported a lower prevalence of postpartum depression (15.6%).^26^ The results of our study are greater than the reported prevalence of postpartum depression in Nairobi, Kenya (13%), Ghana (7%) and a cohort study performed in Ethiopia.^8,27,28^ This could be attributed to the different data collection tools used and the sample sizes of the studies. Nonetheless, in comparison with findings from Egypt (49.5%), South Africa (50.3%) and Nigeria (28%), this study’s level of postpartum depression was lower.^29,30,31^ This could be attributed to the different methodologies used. Among the mothers who had postpartum depression in this study, 18 (24.62%) had thoughts of self-harm. This finding is in line with a finding from an audit study in the United Kingdom (UK) where 24% to 49% of women with prior postpartum depression attempted suicide.^27^ This underscores the need for all stakeholders to accord due attention to postpartum depression, as it poses a potential risk to maternal lives and can have profound effects on the entire household. The morbidity associated with thoughts of self-harm represents an often-unseen burden experienced by affected mothers, even though postpartum depression is preventable and treatable if identified at an early stage. Therefore, it is crucial to diagnose and treat postpartum depression as it could lead to adverse consequences.

This study revealed no significant associations between socio-demographic factors, such as age, occupation and marital status and PPD. However, findings from a study performed in Debre-Berhan, Ethiopia, indicated that postpartum depression was associated with being divorced or widowed and that married women were four times more likely to develop postpartum depression.^25^ A study performed in the Awi zone also revealed that divorced, widowed or single mothers were 3.45 times more likely to develop PPD than those who were married.^28^ Additionally, a study in Uganda also revealed that adolescent mothers were more likely to experience postpartum depression than those in other age groups.^32^ Given the small sample size in this study, the role of socio-demographic factors could have been underestimated.

This study revealed a significant association between unplanned pregnancy and postpartum depression. A systematic review and meta-analysis performed in Ethiopia also revealed a significant association between pregnancy desirability and postpartum depression.^25^ Furthermore, our study revealed a significant association between abortion history and postpartum depression. A community-based study in Northwest Ethiopia also revealed a significant association between PPD and abortion, with mothers who had abortions having a likelihood of experiencing postpartum depression two times greater than those who had never had abortions.^33^ The same study also revealed that respondents who had unplanned pregnancies had a twofold greater risk of experiencing postpartum depression.^33^ Unwanted pregnancies are also a predictor of postpartum depression symptoms in Egypt.^34^ From this finding, the potential psychological impact of abortion can be noted. Hence, this can serve as a ground for further studies to explore the mechanisms of association between abortion history and postpartum depression.

This study revealed no significant associations between the number of pregnancies, mode of delivery and pregnancy complications. There was also no statistically significant correlation between mode of delivery and postpartum depression, according to a systematic review performed in Ethiopia.^25^ However, a study from Nekemte, Oromia, revealed that postnatal depression was 4.99 times more likely to affect first-time mothers than mothers who had given birth more than four times.^35^ Our study revealed that mothers with a history of mental illness were 12 times more likely to develop postpartum depression. A systematic review performed by Tolosa et al. also revealed that women with a prior history of depression were 4.52 times more likely to develop postpartum depression than women without such a history.^25^ Previous depression in mothers increased the likelihood of depression by 3.7 times compared with mothers without such a history in the Awi zone, Ethiopia.^28^ This could be attributed to the biological component of mental illnesses, where there could be a potential predisposition to develop certain conditions.

There was a significant association between the availability of social support and PPD in this study. A study by Tolosa et al. revealed that postpartum depression was 6.59 times more prevalent in women without social support than in those who had good social support.^25^ Similarly, a health facility-based study in Debre-Berhan revealed that mothers with weak social support had a fivefold greater risk of depression than did respondents with good social support.^26^ This could be explained by the substantial effect of the social component of life on mental health.

## Conclusion

Despite the high magnitude of PPD, measures to help depressed mothers are not brought to action. This highlights the need to advocate for postpartum depression services. Although mothers with postpartum depression have made it to the health centre, our study revealed that they have gone back unnoticed. This prevents our health sector from providing comprehensive care to the population. Postpartum depression screening is a simple but profound step that can be implemented during mothers’ visits to vaccination centres. Society-based awareness creation programmes for postpartum depression can be launched so that mothers can be understood and supported during the postpartum period, and this can further help reduce stigma associated with maternal mental health.

Maternal health programmes should integrate routine PPD screening into postnatal care services. Strengthening the capacity of community health workers to identify and refer affected mothers could help improve early diagnosis of postpartum depression. Finally, health professionals can be educated about postpartum depression so that the diagnosis of postpartum depression can be made during multiple encounters of mothers with health facilities.

### Limitations

The taboo against mental health issues in society could have affected the mothers’ responses to the questions during the interviews. Our study is a health facility-based study, which makes it difficult to generalise to the whole population. In addition, recall bias is also a factor that can affect the results of our study. The cross-sectional design of this study also restricts causal inference. The potential influence of social desirability bias is another limitation of this study.

As a result of the lack of response from mothers who were sick and were not able to respond to the questions asked, bias could have been introduced. These mothers may have had a higher likelihood of experiencing depression, which could affect the study’s results. Furthermore, the limited sensitivity and specificity of the EPDS in Ethiopia pose some contextual limitations.
